# Spermatic cord anastomosing hemangioma mimicking a malignant inguinal tumor: A case report and literature review

**DOI:** 10.3389/fsurg.2022.930160

**Published:** 2022-07-22

**Authors:** Zhan-yi Zhang, Peng Hong, Shao-hui Deng, Shi-ying Tang, Zhuo Liu, Hui-ying He, Lu-lin Ma, Shu-dong Zhang, Xiao-jun Tian

**Affiliations:** ^1^ Department of Urology, Peking University Third Hospital, Beijing, China; ^2^ Department of Pathology, Peking University Third Hospital, Beijing, China

**Keywords:** hemangioma, spermatic cord, urogenital neoplasms, hemangiosarcoma, nerve sheath neoplasms

## Abstract

**Background:**

Anastomosing hemangioma (AH) is a rare vascular tumor and occurs in various organs. It is difficult to distinguish AH from malignant tumors even through multimodal imaging examination. AH located in the inguinal region is even rare. We present the diagnosis and treatment of a patient with spermatic cord AH in detail and conduct a literature review.

**Case Report:**

An 84-year-old Chinese man had swelling pain in his right scrotum. A hard and fixed mass was palpable in the right inguinal region. Preoperative radiological examination considered it a neurogenic or vascular tumor. Malignant soft tissue sarcoma could not be excluded. He underwent radical inguinal right orchiectomy under intraspinal anesthesia. The diagnosis of spermatic cord AH was confirmed by pathological examination. The patient recovered uneventfully and remained disease-free during an 18-month follow-up.

**Conclusion:**

Spermatic cord AH is quite rare and could be misdiagnosed as a malignant tumor. Pathological evidence might be necessary. The optimal choice of treatment should be determined through a comprehensive assessment of both tumor and patient factors.

## Introduction

Anastomosing hemangioma (AH) is a rare subtype of vascular tumor. Montgomery and Epstein reported the first AH case in 2009 (1). Thereafter, a number of AH cases have been reported. It seems that AH is usually localized to the genitourinary system, especially in the kidney. Previous reports showed that AH contained a benign course (1–3), with few instances of recurrence or metastasis. However, there are many overlapping clinicopathological features between AH and malignant sarcomas, placing many hurdles to the diagnosis and treatment of AH. AH located in the inguinal region is even rare. In this report, we present a unique case of spermatic cord AH, conduct a brief English literature review about AH, and discuss the differential diagnosis of an inguinal mass according to radiological and pathological features. Besides, reasonable treatment choices are discussed. This study may prove favorable for the diagnosis and treatment of AH at an unusual site.

## Case presentation

**Figure 1:**
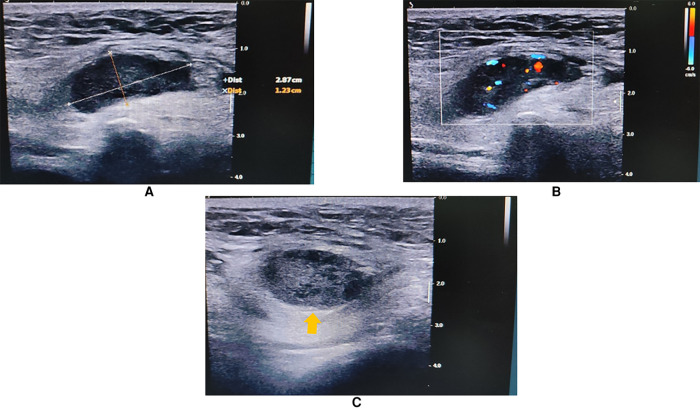
Inguinal US showing a 2.9 ×  1.2 cm solid-cystic mass in the right inguinal region (arrow head). The spermatic cord was swollen with a high echo. (**A**) Long-axis view. (**B**) Color Droppler mode showing blood flow signals within the mass. (**C**) Short-axis view.

An 84-year-old Chinese man came to our clinic complaining of swelling and pain in his right scrotum for 1 week. He could touch a mass in his right inguinal region. Upon physical examination (PE), a 3.0 × 1.5 cm hard and fixed mass could be touched in his right inguinal region. Droppler ultrasound (US) examination was performed initially. A 2.9 × 1.2 cm solid-cystic (predominantly solid) mass could be observed in the right inguinal region. There were abundant blood flow signals within the mass. The spermatic cord was swollen with a high echo. No obvious hernia contents or enlarged lymph nodes were observed when abdominal pressure increased (Figure 1). The radiologists believed that neurogenic tumors could not be excluded and suggested an ultrasound-guided percutaneous biopsy.

**Figure 2:**
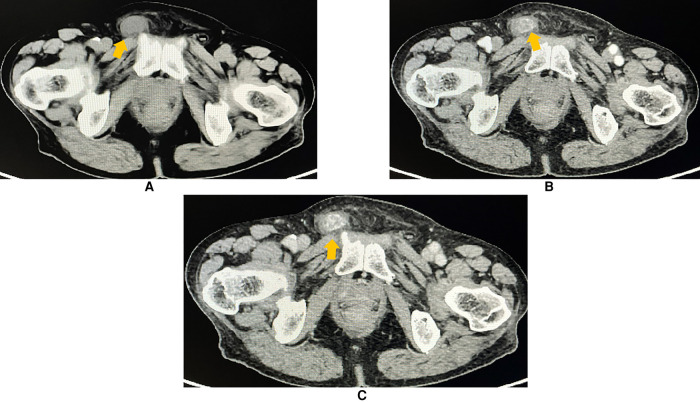
Contrast-enhanced CT (axial plane) revealing a 2.3 ×  1.3 cm heterogeneous and an avidly enhanced mass with a soft-tissue density (arrowhead) in the right inguinal region. Perilesional effusions were observed. (**A**) Non-contrast phase. (**B**) Arterial phase of the contrast. (**C**) Venous phase of the contrast.

**Figure 3:**
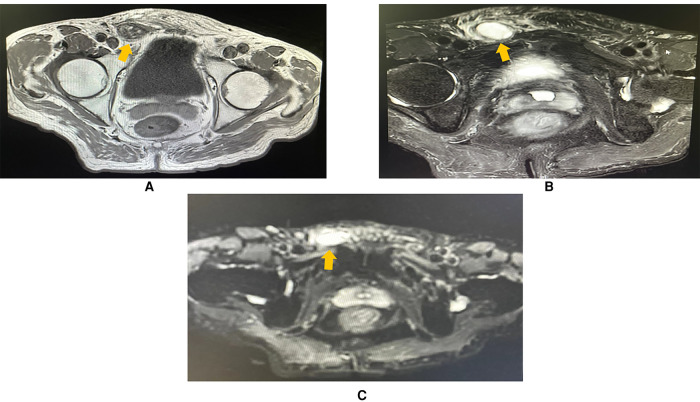
MRI imaging (axial plane) showing a 2.3 ×  1.3 cm oval mass in the right inguinal region, with perilesional effusions and edema. (**A**) T1-weighted imaging showing that the lesion was hypointense (arrowhead). (**B**) T2-weighted imaging showing that the mass (arrowhead) was hyperintense. (**C**) The mass was hyperintense (arrowhead) in diffusion-weighted imaging (DWI).

To learn about the properties, blood supply, and anatomical position of the mass, an abdominal and pelvic contrast-enhanced computed tomography (CE-CT) scan was performed thereafter. A 2.3 × 1.3 cm heterogeneously enhanced mass with soft tissue density was seen inside the right inguinal canal (Figure 2). Hypodense, non-enhanced cystic components could be observed within the mass. To further evaluate the involvement of adjacent soft tissues and lymph nodes, pelvic magnetic resonance imaging (MRI) was then completed. MRI showed a 2.3 × 1.3 cm round-like mass with low signal intensity in T1-weighted imaging (WI) and high signal intensity in T2-weighted imaging (WI). Upon diffusion-weighted imaging (DWI), the mass also presented high signal intensity. Meanwhile, perilesional effusions and edema could be observed. There were no enlarged lymph nodes on both CT and MRI scans (Figure 3). Based on imaging findings, a neurogenic tumor was initially considered by radiologists and might be clinically relevant to his scrotal discomfort. Considering its abundant blood flow, a vascular tumor was also taken into consideration. Moreover, its heterogeneous components and enhancing patterns raised concerns for malignant soft tissue sarcoma. Besides, the patient had a recent history of coronary heart disease (CHD). Therefore, a multidisciplinary team (MDT) discussion with radiologists, pathologists, general surgeons, and cardiologists in our center was conducted. Then, we felt that radical inguinal right orchiectomy might be the appropriate treatment for this patient. We informed the patient about the treatment options, benefits, and potential perioperative risks. He fully understood the benefits and risks of each treatment and chose to undergo surgery.

After intraspinal anesthesia, an oblique incision was made along a Langer line, 2 cm superior and parallel to the right inguinal ligament. The inguinal canal was exposed after incising the external oblique aponeurosis. We transected the cremasteric muscle and exposed the spermatic cord. The mass was palpable within the spermatic cord. The spermatic cord was then dissected bluntly from the mass toward the cephalad direction. Gonadal vessels and vas deferens were separately ligated with absorbable sutures at the deep inguinal ring level. Blunt dissection of the spermatic cord in the caudad direction was performed thereafter. The right testis along with its tunica vaginalis was retracted into the surgical area and completely resected. After careful hemostasis, the fascial layers and skin were sutured. The ilioinguinal nerve was well identified and preserved during the whole surgical procedure.

**Figure 4:**
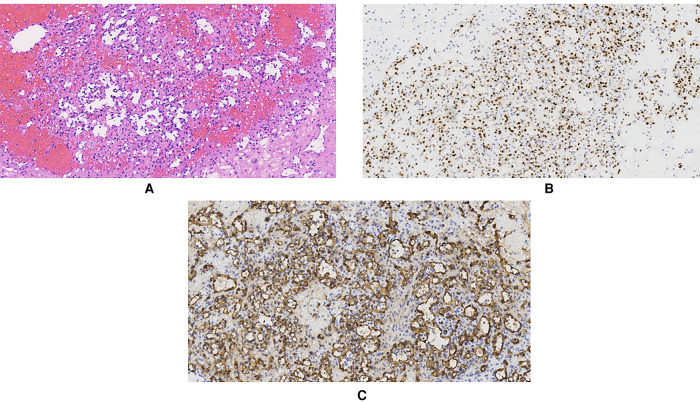
Pathological examination showing that the mass was composed of distinctive “anastomosing” capillary-sized vessels lined by “hobnail” endothelial cells. (**A**) Hematoxylin and eosin (HE) staining (×200). (**B**) Immunohistochemistry staining of ERG in endothelial cells (×200). (**C**) Immunohistochemistry staining of CD34 in endothelial cells (×20).

Grossly, a 1.5 × 1 × 0.8 cm gray-white solid mass could be seen on the sagittal section of the resected specimen. The mass was 1 cm away from the cutting edge of the spermatic cord, 5.5 cm from the testis, and 5 cm from the epididymis. There was no clear boundary between the mass and its adjacent tissues. Microscopically, the mass showed typical features of a vascular tumor. It was composed of irregular, anastomosing capillary-sized vessels. The lumina of those vessels were lined by a monolayer of oval endothelial cells. Some of them showed a hobnail appearance. Those endothelial cells had a mild cellular morphology lacking nuclear atypia and mitoses. Focal short spindle stromal cell hyperplasia could be observed within the spermatic cord. Atrophy in adjacent seminiferous tubules was observed in the right testis. There was no obvious lesion in both resected testis and epididymis. The margin status was negative. Immunohistochemical (IHC) staining showed positive staining of endothelial markers—CD31, CD34, and ERG. Desmin, inhibin-α, S-100, and NSE were negative. The Ki-67 rate was low (approximately 2%) (Figure. 4). SMA was positive in the surrounding stromal cells. Based on the pathological findings, the diagnosis of AH was confirmed.

The patient recovered uneventfully after surgery and was discharged 4 days later. During the 18-month follow-up, the patient reported no local discomfort. PE and abdominal US examination showed no disease recurrence. He expressed satisfaction over the treatment. The timeline of the presented case is summarized in Supplementary Figure S1.

## Discussion

To learn more about this rare subtype of vascular tumor, we searched PubMed, Web of Science, and EMBASE for all AH cases reported in English before September 1, 2021. Fifty-three publications (1–53), including 137 genitourinary cases and 71 non-genitourinary cases, were identified (Supplementary Table S1).

AH patients’ age varies from 1 to 85 years. There are more male patients than females. Most genitourinary AHs locate in the kidney, while the para-vertebral region is the most frequently involved region in non-genitourinary patients (Supplementary Figure S2A). The tumor size ranges from 0.1 to 14.0 cm and tends to be larger in non-genitourinary patients (Supplementary Figure S2B). More than half of AH cases are incidentally found in routine examination. The common manifestations are local pain, palpable mass, hematuria, neurological deficit, hematoma, and so on (Supplementary Figure S2C). Moreover, 21.7% of genitourinary cases and 11.3% of non-genitourinary cases are coexistent with other tumors (Supplementary Figure S2D). In this article, we presented an 84-year-old man with a rare, symptomatic spermatic cord AH. The patient complained of discomfort in his scrotum, and a palpable mass was palpable in his right inguinal region. It could be deducted that the symptom in the scrotum might be caused by the inguinal mass. It is reported that spermatic cord masses are generally benign, and inguinal lipoma is the most common tumor. However, the incidence of malignancy is not low, which could reach up to 30% (54). It is also noteworthy that about 46% of soft tissue sarcoma is located in the thigh, buttock, and inguinal regions (55). Thus, although the incidence of sarcoma is only about 2/100,000 in China (56), we should still make a careful differential diagnosis of this inguinal mass.

Currently, there is a lack of specific imaging features for a definite diagnosis of AH, but some common presentation is already confirmed. AHs usually show a hypoechoic or anechoic cystic lesion on US (6, 20, 26, 57) and may have an enrichment of blood flow on edge (21). Nearly all AH patients underwent a regional CT examination. On CT scans, the majority of AHs are well-circumscribed, hyperdense nodules with or without centrally hypodense cystic components. The existence of post-contrast avid peripheral enhancement and the filing pattern of contrast agent from the peripheral region to the center could reveal the properties of hemangiomas (2, 36, 37). Some patients underwent MRI scans with or without gadolinium enhancement (3, 5, 26, 34, 36–38). AHs tend to be hypointense on T1WI and hyperintense on T2WI and DWI. Peripheral enhancement in the arterial phase and enhancement of central components in the venous phase could be observed, similar to the manifestations of CE-CT (5).

Our patient showed a hypoechoic mass with a prominent blood circulation signal on US. The mass was peripherally isodense with centrally hypodense cystic components on non-contrast images and could be heterogeneously and avidly enhanced. Along with its characteristics on MRI (hypointense T1 signal, hyperintense T2, and DWI signal), it showed similar imaging features to the previously reported AHs, while it lacked the typical filing pattern of hemangiomas on CE-CT. Radiologists from our center considered the initial diagnosis of neurogenic tumors such as schwannoma. Schwannoma is a benign peripheral nerve sheath tumor (PNST), which may also present as a hypoechoic cystic mass on US. On MRI, schwannoma shows hypo- or iso-intensity, compared with adjacent muscles on T1WI and high intensity on T2WI, especially on the periphery. Cystic degeneration may also exist on CT/MRI. However, typical characteristics, such as nerve entering or existing the mass, “fusiform” shape, “split-fat” sign, and “target” sign, were absent in our case (58, 59). Moreover, imaging signs, including predominant solid components, rich blood supply, perilesional effusions, and a heterogeneous enhancement pattern implied the possibility of a soft tissue sarcoma, for example, malignant PNST. Thus, pathological examination seemed necessary for the diagnosis.

There exist controversial opinions on the optimal way to acquire pathological specimens in this circumstance. According to a literature review, all genitourinary AH cases underwent surgery, while some non-genitourinary cases chose relatively conservative treatment (Supplementary Figure S2E). Image-guided percutaneous biopsy provides a less traumatic choice (36), and the outcome could help decide subsequent therapies. However, the limited histologic materials acquired from a percutaneous biopsy may be challenging to make an accurate diagnosis, especially when the mass shows heterogeneous entities. Results from surgically acquired specimens might be more reliable, while the extent of resection remains to be discussed. A literature review on the surgical management of spermatic cord sarcoma (60) and an MDT discussion were conducted. There were several factors in favor of radical inguinal orchiectomy: First, the mass had no clear border with adjacent spermatic cord vessels and was rich in blood supply, which made sole dissection rather difficult. Second, considering that the mass might be malignant and that no metastatic sites were noted, surgical margin status would be maximally guaranteed and the possibility of reoperation as well as local recurrence might be reduced. Last but not least, our patient was an 84-year-old and did not have fertile demands. The pursuit of R0 resection of a susceptible malignant inguinal mass was acceptable. In consideration of the patient's condition and preference, a radical inguinal right orchiectomy at the level of the deep inguinal ring was performed.

According to the literature, the majority of AHs have a clear margin but lack a definite capsule and usually present a hemorrhagic “mahogany brown” (47) or “red-tan spongy” (1) gross appearance. Microscopically, the tumors are composed of “anastomosing” capillary-sized vessels resembling the splenic sinusoids. The vascular channels within the tumor are capillary-sized, lined by a single layer of endothelial cells that frequently shows a hobnail morphology (1, 11, 39, 42). Edematous or myxoid components may exist in the surrounding stroma (25, 61). Extramedullary hematopoiesis and intraluminal thrombi are occasionally observed (10, 32, 45). Some uncommon features, such as focally infiltrative patterns (6, 46) and hyaline globules (18, 28), might raise concerns about some aggressive malignant tumors, such as angiosarcoma and Kaposi sarcoma. Differential diagnosis of well-differentiated low-grade angiosarcoma required the most careful recognition (39). However, AHs lack malignant signs like significant endothelial tufting, nuclear atypia, pleomorphism, mitosis, and multilayers of endothelial cells. These may help morphological discrimination through careful observation (13, 39). In IHC staining, AHs are typically positive for endothelial markers like CD31, CD34, Factor VIII, and FLI1, while they usually lack the immunoactivity of lymphatic endothelial cell marker D2-40, splenic sinusoidal cell marker CD8, and immunosuppressive-related tumor marker HHV8 (62).

Our patient presented a gross gray-white appearance, which was distinct from the reported AHs. Rather than a neurogenic tumor, the microscopic histological features revealed typical features of an endothelial vascular tumor reminiscent of spleen sinusoids, and malignant morphologic signs of angiosarcoma were absent. CD31, CD34, and ERG were positive in IHC staining. ERG is also an endothelial marker, but it is more specific and sensitive than CD31 or CD34. ERG (+) has also been reported previously in AH patients (15, 16, 30, 40, 44, 53). As spindle stromal cell hyperplasia was observed within the spermatic cord, we stained the markers of spindle cell sarcoma (e.g., rhabdomyosarcoma, leiomyosarcoma, regressed germ cell tumors, and MPNST), such as desmin, inhibin-α, S-100, and NSE. They all showed negative results. Thus, the diagnosis of a rare spermatic cord AH was eventually confirmed.

Overall, AHs have a benign course. In the literature review, follow-up time ranges from 0.5 to 156 months. Few instances of recurrence or metastasis were reported, except for two unique cases. One was an intracranial AH (10), and the other one had multifocal, recurrent AHs located at different sites (12). Three patients died from unrelated disease. No disease-related death was reported. These results imply that there might be no need for further therapies when the diagnosis of AH is confirmed. If the patient was diagnosed with surgical resection, negative margin status could minimize the risk of local recurrence. Regular follow-up is still recommended, especially for those diagnosed with percutaneous biopsy or for those choosing active surveillance. As for the pathogenesis of AH, recent studies have found that recurrent GNA11, GNA14, and GNAQ gene mutation occurs in some AH cases, which is different from that of angiosarcoma (7, 8, 28). Mutational analysis may serve as a tool of differential diagnosis in the future.

There exist some limitations in the current study. IHC staining of some markers like D2–40, GLUT-1, and HHV-8, which may help to exclude lymphatic tumors and other vascular tumors, was absent. The follow-up time is not so long to evaluate recurrence and tumor-specific survival.

## Conclusions

Spermatic cord AH is quite rare and could be misdiagnosed as a malignant inguinal tumor. Pathological examination is necessary when the diagnosis cannot be made through multi-modal radiology assessment. The optimal choice of treatment should be determined through a comprehensive assessment. If the mass has a high risk of malignancy and the patient may not benefit from percutaneous biopsy, a meticulously tailored surgery might be a reasonable choice.

## Data Availability

The raw data supporting the conclusions of this article will be made available by the authors, without undue reservation.
