# Supplementary material for: Spermatic cord anastomosing hemangioma mimicking a malignant inguinal tumor: A case report and literature review
Source: Front Surg. 2022 Jul 22;9:930160. doi: 10.3389/fsurg.2022.930160 (PMC9354528; doi:10.3389/fsurg.2022.930160)
Supplement: Supplementary file 1 [file Table_1_v1.docx]

| **Author, year** | **Location** | †**case number** | **Age (yrs)** | **Sex (Male/Female)** | **Treatment（1=percutaneous biopsy, 2=surgical resection）** | **Follow-up time (mo)** |
| --- | --- | --- | --- | --- | --- | --- |
| **Genitourinary cases** | | | | | | |
| Montgomery E et al, 2009 | Kidney | 4 | 49-75 | 2/2 | 2 | 8-36, NA(n=1) |
|  | Testis | 2 | 49,54 | 2/0 | 2 | 8,12 |
| Brown JG et al, 2010 | Kidney | 5 | 21-83 | 2/3 | 2 | 24-72, NA(n=2) |
| Kryvenko ON et al, 2011 | Kidney | 5 | 39-67 | 1/4 | 2 | 3-122 |
|  | Ovary | 3 | 49-77 | 0/3 | 2 | 1-32 |
| Mehta V et al, 2012 | Kidney | 3 | 45-55 | 3/0 | 2 | 3-12 |
| Ross M et al, 2012 | Adrenal Gland | 1 | 49 | 1/0 | 2 | NA |
| Tran TA et al, 2012 | Kidney | 1 | 59 | 1/0 | 2 | NA |
| Büttner M et al, 2013 | Kidney | 8 | 32-69.8 | 6/2 | 2 | NA |
| Wetherell DR et al, 2013 | Kidney | 1 | 74 | 1/0 | 2 | 0.75 |
| Zhao M et al, 2013 | Kidney | 1 | 48 | 1/0 | 2 | 12 |
| Chou S et al, 2014 | Kidney | 2 | 50,60 | 1/1 | 2 | 14,8 |
| Heidegger I et al, 2014 | Kidney | 1 | 56 | 1/0 | 2 | 156 |
| Kryvenko ON et al, 2014 | Kidney | 15 | 15-68 | 14/1 | 2 | NA |
| Tao LL et al, 2014 | Kidney | 1 | 32 | 1/0 | 2 | 21 |
| Tahir M et al, 2015 | Kidney | 1 | 57 | 1/0 | 2 | 1 |
| Omiyale AO et al, 2015 | Kidney | 1 | 64 | 1/0 | 2 | 10 |
| Zhang W et al, 2015 | Kidney | 1 | 29 | 0/1 | 2 | 16 |
| Jin LU et al, 2016 | Bladder | 1 | 46 | 1/0 | 2 | NA |
| O'neill AC et al, 2016 | Kidney | 11 | NA | NA | 2 | NA |
|  | Adrenal Gland | 1 | NA | NA | 2 | NA |
|  | Ovary | 4 | NA | 0/4 | 2 | NA |
|  | Testis | 1 | NA | 1/0 | 2 | NA |
|  | Spermatic cord | 1 | NA | 1/0 | 2 | NA |
| ‡John I et al, 2016 | Ovary | 1 | 74 | 0/1 | 2 | NA |
|  | Uterus | 1 | 36 | 0/1 | 2 | NA |
| Bean GR et al, 2017 | Uterus | 1 | 70 | 0/1 | 2 | NA |
|  | Ovary | 2 | 60,65 | 0/2 | 2 | 20, NA |
|  | Kidney | 3 | 49-64 | 3/0 | 2 | 9-107 |
|  | Adrenal Gland | 1 | 39 | 1/0 | 2 | 12 |
| Abboudi H et al, 2017 | Kidney | 2 | 62,62 | 0/2 | 2 | NA |
| Al-Maghrabi HA et al, 2017 | Kidney | 1 | 55 | 0/1 | 2 | 3 |
| Dundr P et al, 2017 | Ovary | 6 | 43-81 | 0/6 | 2 | 3-52, NA(n=2) |
| Berker NK et al, 2017 | Kidney | 2 | 24,57 | 0/2 | 2 | 10,4 |
| Burton KR et al, 2017 | Kidney | 1 | 68 | 1/0 | 2 | 24 |
|  | Adrenal Gland | 1 | 68 | 1/0 | 2 | 24 |
| Perdiki M et al, 2017 | Kidney | 2 | 47,64 | 1/1 | 2 | 25,14 |
| Silva MA et al, 2017 | Kidney | 1 | 53 | 2/0 | 2 | NA |
| Bean GR et al, 2018 | Ovary | 1 | 60 | 0/1 | 2 | NA |
|  | Adrenal Gland | 1 | 42 | 1/0 | 2 | NA |
| Cheon PM et al, 2018 | Kidney | 1 | 40 | 1/0 | 2 | 1 |
| Kishida N et al, 2018 | Kidney | 1 | 75 | 0/1 | 2 | NA |
| Gunduz M et al, 2019 | Ovary | 1 | 62 | 0/1 | 2 | NA |
| Subbarayan D et al, 2019 | Ovary | 1 | 50 | 0/1 | 2 | NA |
| Caballes AB et al, 2019 | Kidney | 1 | 10 | 1/0 | 2 | NA |
| Patel SR et al, 2019 | Kidney | 2 | 39,39 | 2/0 | 2 | NA |
|  | Adrenal Gland | 1 | 39 | 1/0 | 2 | NA |
| Liau JY et al, 2020 | Kidney | 5 | 23-70 | 3/2 | 2 | NA |
|  | Ureter | 1 | 60 | 0/1 | 2 | NA |
|  | Ovary | 2 | 45,51 | 0/1 | 2 | NA |
|  | Uterus | 1 | 43 | 0/1 | 2 | NA |
| Rezk A et al, 2020 | Ovary | 1 | 60 | 0/1 | 2 | NA |
| Stewart CJR et al, 2020 | Ovary | 1 | 48 | 0/1 | 2 | NA |
| Manohar V et al, 2020 | Kidney | 1 | 40 | 0/1 | 2 | 24 |
| Zhou J et al, 2020 | Kidney | 7 | NA | NA | 2 | NA |
|  | Adrenal Gland | 3 | NA | NA | 2 | NA |
| Zheng LP et al, 2020 | Kidney | 1 | 74 | 0/1 | 2 | 2 |
| Lo CH et al, 2021 | Kidney | 1 | 84 | 1/0 | 2 | 0.5 |
| **Non-genitourinary cases** | | | | | | |
| Lin J et al, 2013 | Liver | 4 | 48-71 | 0/2 | 2 | 67,14 |
|  | Gastrointestinal tract | 2 | 68,70 | 1/1 | 2 | NA,8 |
| *John I et al, 2016 | Para-vertebral region | 13 | 31-85 | 11/2 | 1(n=7) 1+2(n=1) 2(n=5) | 1-46, NA(n=4) |
|  | Mediastinum | 1 | 70 | 0/1 | 2 | 1 |
|  | Limb | 1 | 2 | 0/1 | 2 | 1 |
| O'neill AC et al, 2016 | Retroperitoneum (NOS) | 7 | NA | NA | NA | NA |
|  | Para-vertebral region | 4 | NA | NA | NA | NA |
|  | Liver | 2 | NA | NA | NA | NA |
|  | Gastrointestinal tract | 1 | NA | NA | NA | NA |
| Bean GR et al, 2017 | Peritoneum | 1 | 58 | 1/0 | 2 | 12 |
|  | Para-vertebral region | 4 | 53-79 | 3/1 | 2 | 1-40 |
|  | Liver | 1 | 66 | 1/0 | 2 | NA |
| Burton KR et al, 2017 | Para-vertebral region | 1 | 68 | 1/0 | 2 | 24 |
| Peng X et al, 2017 | Liver | 1 | 57 | 0/1 | 2 | 12 |
| Jayaram A et al, 2018 | Para-vertebral region | 1 | 53 | 0/1 | 2 | NA |
| Gonzalez SP et al, 2019 | Liver | 1 | 62 | 0/1 | 2 | 18 |
| Lunn B et al, 2019 | Liver | 5 | 33-77 | 3/2 | 1(n=2) 1+2(n=1)2(n=2) | 36 |
| Merritt B et al, 2019 | Liver | 1 | 56 | 1/0 | 1 | NA |
| Tran TAN et al, 2019 | Limb | 1 | 41 | 0/1 | 2 | NA |
| Bodman A et al, 2020 | Cerebrum | 1 | 33 | 1/0 | 2 | NA |
| Dutta R et al, 2020 | Larynx | 1 | 37 | 1/0 | 2 | 12 |
| Huang ZY et al, 2020 | Nasal cavity | 1 | 37 | 1/0 | 2 | 22 |
| Liau JY et al, 2020 | Retroperitoneum (NOS) | 3 | 70 | 1/2 | 2 | NA |
|  | Para-vertebral region | 4 | 33-79 | 2/2 | 2 | NA |
|  | Mediastinum | 2 | 53,61 | 1/1 | 2 | NA |
|  | Axilla | 1 | 56 | 0/1 | 2 | NA |
|  | Sub-phrenic region | 1 | 71 | 1/0 | 2 | NA |
|  | Liver | 2 | 62 | 0/1 | 2 | NA |
|  | Gastrointestinal tract | 1 | 53 | 0/1 | 2 | NA |
| Lin MS et al, 2020 | Breast | 1 | 49 | 0/1 | 2 | 5 |
| Rathore K et al, 2020 | Pericardium | 1 | 64 | 1/0 | 2 | NA |

NOS, Not Otherwise Specified

†Multifocal lesions in the same organ were regarded as one case, while bilateral lesions were considered as two cases.

‡Clinical features of most cases in this literature were absent, and thus only locations of these cases were included in our review.
