# Supplementary figures and images for: Spermatic cord anastomosing hemangioma mimicking a malignant inguinal tumor: A case report and literature review
Source: Front Surg. 2022 Jul 22;9:930160. doi: 10.3389/fsurg.2022.930160 (PMC9354528; doi:10.3389/fsurg.2022.930160)

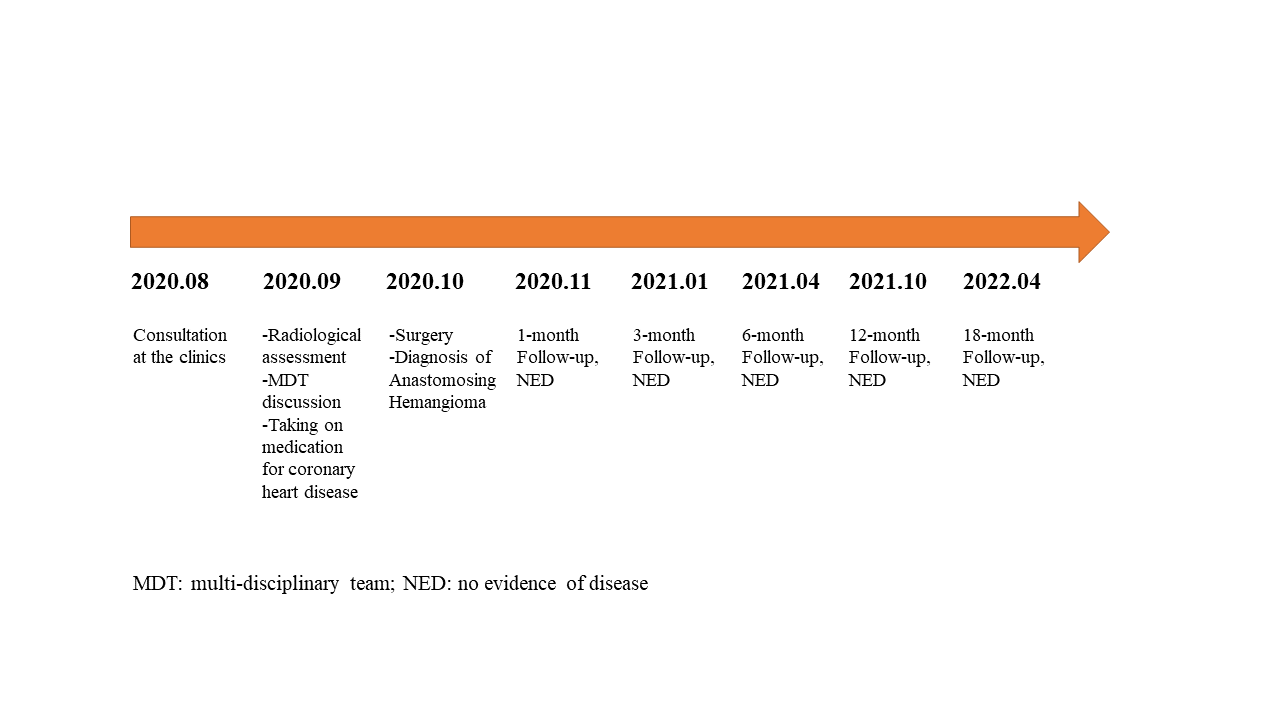

Supplement: Supplementary file 2 [file Image_1_v1.tiff]

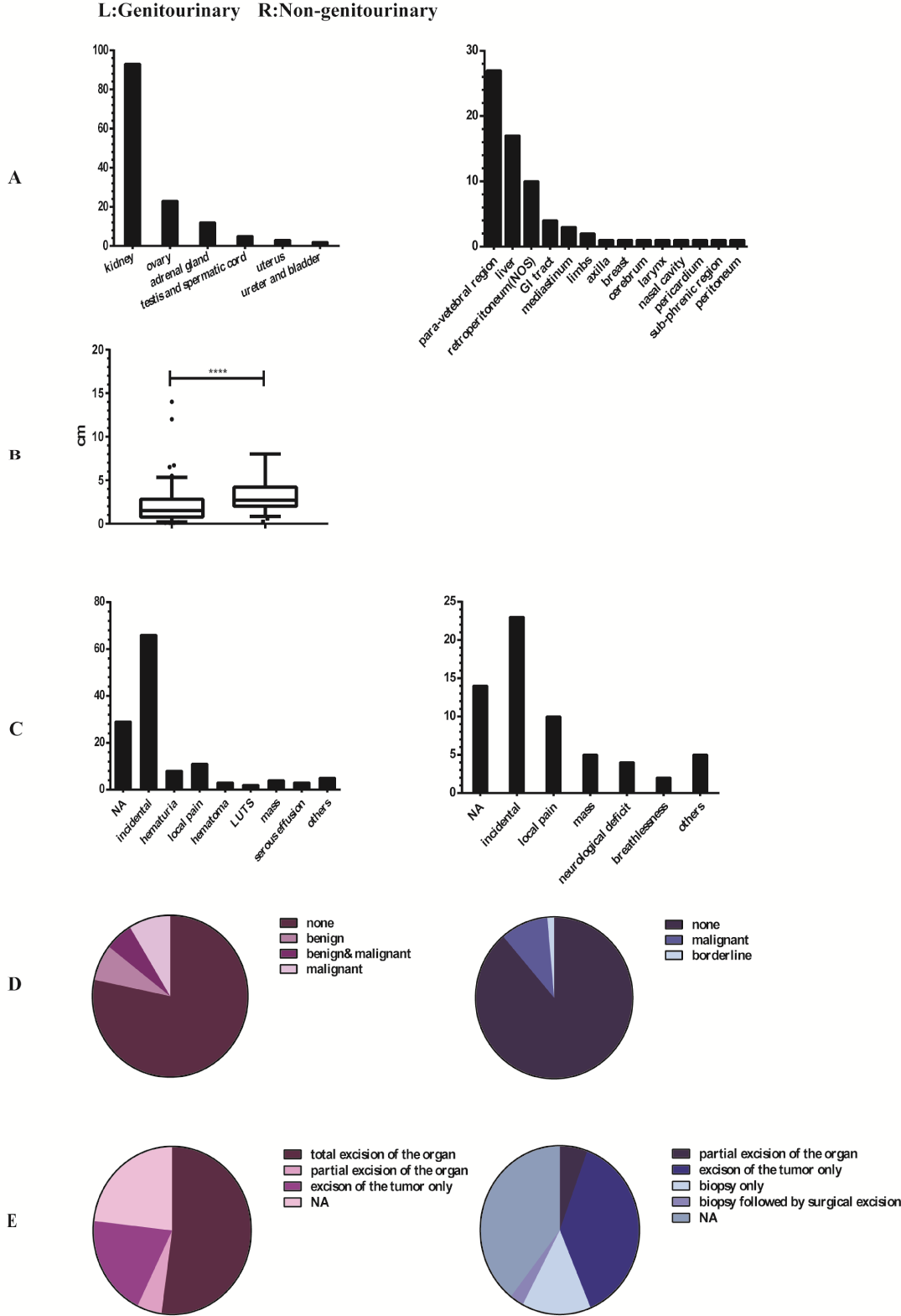

Supplement: Supplementary file 3 [file Image_2_v1.tiff]
